# Manipulating Bodily Presence Affects Cross-Modal Spatial Attention: A Virtual-Reality-Based ERP Study

**DOI:** 10.3389/fnhum.2017.00079

**Published:** 2017-02-22

**Authors:** Ville J. Harjunen, Imtiaj Ahmed, Giulio Jacucci, Niklas Ravaja, Michiel M. Spapé

**Affiliations:** ^1^Helsinki Institute for Information Technology, Department of Computer Science, Aalto UniversityEspoo, Finland; ^2^Social Psychology, Department of Social Research, University of HelsinkiHelsinki, Finland; ^3^Helsinki Institute for Information Technology, Department of Computer Science, University of HelsinkiHelsinki, Finland; ^4^Information and Service Economy, School of Business, Aalto UniversityHelsinki, Finland; ^5^Helsinki Collegium for Advanced Studies, University of HelsinkiHelsinki, Finland; ^6^Department of Psychology, Liverpool Hope UniversityLiverpool, UK

**Keywords:** virtual reality, head mounted display, event-related potentials, bodily presence, cross-modal spatial attention

## Abstract

Earlier studies have revealed cross-modal visuo-tactile interactions in endogenous spatial attention. The current research used event-related potentials (ERPs) and virtual reality (VR) to identify how the visual cues of the perceiver’s body affect visuo-tactile interaction in endogenous spatial attention and at what point in time the effect takes place. A bimodal oddball task with lateralized tactile and visual stimuli was presented in two VR conditions, one with and one without visible hands, and one VR-free control with hands in view. Participants were required to silently count one type of stimulus and ignore all other stimuli presented in irrelevant modality or location. The presence of hands was found to modulate early and late components of somatosensory and visual evoked potentials. For sensory-perceptual stages, the presence of virtual or real hands was found to amplify attention-related negativity on the somatosensory N140 and cross-modal interaction in somatosensory and visual P200. For postperceptual stages, an amplified N200 component was obtained in somatosensory and visual evoked potentials, indicating increased response inhibition in response to non-target stimuli. The effect of somatosensory, but not visual, N200 enhanced when the virtual hands were present. The findings suggest that bodily presence affects sustained cross-modal spatial attention between vision and touch and that this effect is specifically present in ERPs related to early- and late-sensory processing, as well as response inhibition, but do not affect later attention and memory-related P3 activity. Finally, the experiments provide commeasurable scenarios for the estimation of the signal and noise ratio to quantify effects related to the use of a head mounted display (HMD). However, despite valid a-priori reasons for fearing signal interference due to a HMD, we observed no significant drop in the robustness of our ERP measurements.

## Introduction

Our ability to focus on a specific location while ignoring events occurring in other directions is a vital requirement for successful interaction with the surrounding world. While early research on selective spatial attention focused on attention processes within a single sensory modality (Woods, 1990), over the last two decades evidence has cumulated on the degree that voluntary—or endogenous—attention in one modality and spatial location strongly affects processing in the other, task-irrelevant, sensory modalities if presented on the attended side (Spence, 2014). For example, in a study by Spence et al. (2000), asking participants to respond to visual stimuli presented at certain location was shown to speed up participants’ reactions to visual and tactile events if presented on the attended side.

Recordings of ERPs have been found particularly useful in the investigation of the mechanisms underlying endogenous cross-modal spatial attention. A typical ERP experiment involves a stream of stimuli presented in two sensory modalities and from two spatial locations (e.g., left and right). Participants are asked to respond to stimuli of a certain combination of modality and location while ignoring all other stimuli. The general finding is that attending to a location amplifies evoked potentials in the target modality and in the irrelevant modality (Eimer, 2001). In other words, even if the participants should completely ignore stimuli in the irrelevant modality and merely respond to, for example, left vibrations, visual ERPs show enhanced processing if they appear in the relevant location (left). Such cross-modal interactions have been observed in various modalities: as suggested between vision and audition, as well as between vision and audition and audition and touch (Teder-Sälejärvi et al., 1999; Eimer et al., 2002).

In EEG-based ERP measurements, the effect of spatial attention usually occurs in the sensory-specific N1 component, suggesting the modulation takes place at a very early, sensory-perceptual stage (Hötting et al., 2003). This observation with the findings that preparatory attentional states are similarly affected regardless of the target modality, have led researchers to conclude that endogenous spatial attention operates at a supramodal level (Eimer et al., 2002). That is, rather than being divided into separate unimodal attention systems, our spatial selection seems to be regulated by a modality independent control system operating across different sensory systems.

Cross-modal links also have been demonstrated in completely different settings, demonstrating the special role of the visual body in spatial attention. Sambo et al. (2009), for instance, investigated whether the visual input of one’s hands would influence the somatosensory processing of tactile targets in a sustained spatial attention task. Participants were instructed to covertly attend to infrequent tactile targets presented to one hand while completely ignoring targets sent to the other hand, as well as all non-targets. Attending to the stimulated hand was found to enhance early somatosensory processing. The attentional modulation, however, occurred earlier (from 100 ms poststimulus) if the participant’ hands were visible when they were covered, or if the participant was blindfolded. Similarly, a positron emission topography study by Macaluso et al. (2000) revealed that covertly attending to one’s left or right hand resulted in greater activity in intraparietal sulcus and secondary somatosensory cortex when a tactile stimulus was delivered to the attended hand. Finally, a more recent study on this visual enhancement of touch (VET) showed that viewing one’s body affected processing of the tactile stimuli and that the effect was observed as early as 27 ms poststimulus in primary somatosensory cortex (Longo et al., 2011).

Thus, it is clear that seeing our body affects even the earliest levels of perceptual processes (Harris et al., 2015). Previous findings suggest, however, that attentional modulation can also affect later stages of processing, such as response inhibition and execution. Pavani et al. (2000), for instance, examined the attentional link between vision and touch using a spatial tactile discrimination task with visual distractors. In all conditions, the visual distractors were presented well away from participants’ hands, which, in turn, were occluded from view by a table. Reaction times in the spatial discrimination task were substantially delayed if the visual distractors were spatially incongruent with the responses. Interestingly, however, the researchers found even greater delay if placing a pair or rubber hands close to the distractors, suggesting the visual body, whether rubber or real, is used to locate events in the tactile modality. To some extent, this is similar to the VET (Sambo et al., 2009; Longo et al., 2011) but there are also crucial differences. First, in the VET studies, the visual body cues were shown to enhance *tactile* perception while in Pavani et al.’s (2000) study, visual input of the hands enhanced processing of *visual* stimuli. Second, in the VET, the visual body input mainly affected the early sensory–perceptual processes while the Pavani et al.’s (2000) results indicate seeing one’s hands influenced later executive functions, such as response inhibition.

Unfortunately, Pavani et al. (2000) did not investigate physiological responses, leaving us to speculate about the brain stages affected by the rubber hand effect. Taking into account that reaction times are commonly correlated with the P3 potential (Conroy and Polich, 2007), one could expect the tactile target-related P3 to be more enhanced if one’s hands are visible than when they are occluded. Similarly, if seeing a visual distractor close to one’s hands makes it more distracting, as Pavani et al. (2000) suggested, one would predict that more of an inhibitory effort would be required if the hands are visually present than when they are not. Because the anterior N2 component—a negative potential occurring just before the P300—has been found to be particular to inhibitory processes (Spapé et al., 2011; for a review, see Folstein and Van Petten, 2008), one could predict the visual presence of hands may result in an amplified anterior N2. On the other hand, earlier research on cross-modal spatial attention (for review, see Eimer et al., 2002) shows the cross-modal interactions are clearly present in early sensory processes while completely absent in late postperceptual processes. It is thus possible the modulating effect of bodily presence is likewise limited to sensory-related components, such as the N1, which has been linked to early sensory gain control that amplifies spatially relevant sensory stimuli (Hillyard et al., 1998b), and P200, which has been thought to reflect the attentional enhancement of late sensory processing (Freunberger et al., 2007).

Thus, the goals of the current ERP study were to verify whether seeing one’s body affects the spatial attentional link between vision and touch and to determine at which point the effect takes place. Similar to earlier studies, we utilized a bimodal oddball paradigm with two sensory modalities and two locations leading to four stimulus types: left- and right-handed vibrations and left- and right-located flashes. Participants reacted to one stimulus type by silently counting the occurrences while ignoring all other stimuli arising from the other modality and direction. The effect of bodily presence on cross-modal spatial attention was then investigated by manipulating participants’ visual body cues under three viewing conditions. In the *VR hands* condition, motion tracking sensors, and a head mounted display (HMD) were used to show the task in a virtual scenario with simulated hands. In the *VR without hands* condition, no virtual hands were provided, leaving the participant virtually disembodied in visual space. Finally, a control condition was provided with the task shown in the traditional setup without any VR. To determine whether and when the bodily presence affects cross-modal interaction, we measured the effect of visible hands on the attentional modulation of visual and tactile evoked N1, P2, N2, and P3 potentials, contrasting the virtual hand condition with both a no-hand VR condition and a VR-free control condition.

Further, we were interested in measuring the effect of using an HMD on the reliability of the EEG signal. Given the anecdotal findings of HMDs’ adverse influences on ERPs (Bayliss and Ballard, 2000), we took special care to make conditions between the different scenarios commeasurable and to quantify the degree to which the HMD induces noise in the EEG signal. To do so, we concentrated on the most common ERP, the P3, and compared the signal to noise results obtained in VR conditions with equivalent ERP data collected in traditional experimental settings with an LCD screen (HMD-free control). After taking into account that the type of stimulus and the location of the experiment affects ERPs (Debener et al., 2012), we created a virtual replica of our real laboratory environment, including the EEG amplifier and other objects, such as the LCD screen. As the participants put on the HMD, they found themselves in seemingly the same room, but now in VR. Finally, the same experimental procedure was used to project the stimuli on the real screen in the control condition as it appeared on the virtual animated screen. Thus, additional noise could not be ascribed to differences in the scenario or task and must instead be due to the HMD itself.

## Materials and Methods

### Participants

Twelve right-handed participants (seven female, five male) volunteered to take part in the experiment. They all self-reported as healthy adults (age 29.5 ± 5.7 years) with normal vision. In accordance with the Declaration of Helsinki, participants were fully briefed on the nature of the study and on their rights as volunteers—including the right to withdraw at any point without fearing negative consequences—prior to signing informed consent forms. The order of the experimental conditions was counterbalanced among participants so that one-third of the participants took part in the control condition (real hands) first. After the three conditions, the participants received two movie tickets in return for their participation. The study did not concern medical research, and in accordance with Finnish law, the need for formal approval was waived by both the vice president of Aalto University and by the chairman of the Ethics Review Board of Aalto.

### Procedure and Design

**FIGURE 1:**
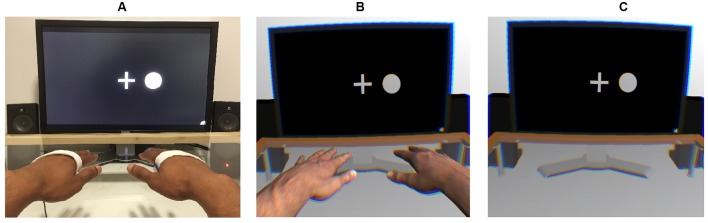
**Experimental setups used in the control (A)**, virtual-hand **(B)**, and no-hand **(C)** conditions. An EEG was recorded while a stream of tactile and visual stimuli was presented on the left and right sides. Participants were instructed to place their hands on the table in alignment with the visual stimuli.

A 3 (viewing condition: control [HMD-free], VR with hands, VR without hands) × 24 (blocks) × 4 (target stimulus: vibration on the left, vibration on the right, circle on the left, circle on the right) × 4 (presented stimulus: vibration on the left, vibration on the right, circle on the left, circle on the right) within-subject design was employed. **Figure 1** shows the setups of each viewing condition.

In the Oddball task, participants were instructed to silently count a certain stimulus type (e.g., flashes on the left) while a stream of tactile and visual stimuli was presented on both the left and right sides of a central fixation cross. The cross was presented at the center of the screen throughout the stream of stimuli, and participants were told to keep their gaze on it while counting the stimuli. Following the block, participants were asked to indicate the correct number of the target stimuli. The task was the same in all three viewing conditions (control, VR with hands, and VR without hands, order counterbalanced among participants). Each condition consisted of 24 blocks of 60 stimulus trials. All four stimulus types (vibrations on left, vibrations on right, flashes on left, and flashes on right) were presented with equal probability of 25% within the block. However, to keep participants from guessing the correct answer, the number of target stimuli was randomly varied (15 ± 3) among blocks.

Each block proceeded uniformly, beginning with a task instruction presented on the screen (e.g., “Count the flashes left”) for 3 s, after which the white central fixation cross was shown on a black background. Following an interval of 100–300 ms (randomized), the stream of stimuli started with a 500-ms stimulus duration and 100–300-ms (randomized) inter-stimulus interval. The next block started immediately after participants indicated the number of targets. The entire experiment took approximately 100 min, including breaks and EEG preparation.

### Apparatus and Stimulus Material

In all three viewing conditions, participants were seated at a desk equipped with a glass table. Vibrotactile devices were placed on each participant’s left and right palms and kept stationary with rubber bands. Vibrations were presented using two ATAC C-2 Tactors^1^ that each delivered 125-Hz sinusoid signals with a 500-ms stimulus duration. To prevent the C-2 from providing auditory cues, masking white noise was played throughout the experiment.

The source of the visual stimuli differed depending on the viewing condition. In the control condition, the visual stimuli and task instructions were presented on a 24′′ TFT monitor (1920 pixel × 1200 pixel resolution; 60 Hz refresh rate), whereas in the VR conditions, an Oculus Rift VR headset (Oculus Rift Developer Kit 2; 960 × 1080 resolution per eye; 75 Hz refresh rate; 100° nominal field of view) was used. However, in all conditions, the visual stimuli that had to be counted were white, filled circles (200 pixels/5.4 cm diameter) presented for 500 ms to the left or right of a central fixation cross. The validity of comparing the control with the VR conditions was further supported by adding a photorealistic 3-D model of the lab room to the VR setting, including all of the physical lab’s central visual objects (e.g., computer screen, amplifier, speakers, and a glass table; see Supplementary Figure 2). No visual body cues were present in the first VR condition. However, to investigate how bodily presence affects cross-modal spatial attention, a pair of virtual arms—the appearance of which matched participants’ real arms—was included in the second VR condition. To allow participants to move their 3-D arms in the virtual space, we placed a Leap Motion^2^ movement tracker under the glass table 16 cm below each participant’s real hands.

The stimuli timing and the behavioral data recording were enabled via the Unity3D platform (Unity Technologies, San Francisco, CA, USA; version 4.6.9), using custom C#-programmed routines to facilitate timing accuracy and sending triggers via parallel port to the EEG amplifier. The same experimental code was used in all viewing conditions to present the visual and tactile stimuli. Integration with the HMD was achieved using the Oculus Unity Integration Package (Oculus VR, Irvine, CA, USA; version 0.4.3). Finally, all conditions were presented using the same Intel desktop PC, which ran Windows 7.

### EEG Recording and Preprocessing

A QuickAmp amplifier (Brain Products GmbH, Gilching, Germany) recorded the EEG at 1,000 Hz from 32 Ag/AgCl scalp electrodes, which were positioned using an elastic EEG cap (EasyCap GmbH, Herrsching, Germany) on the approximately equidistant electrode sites of the 10% system. The initial recording reference was the common average reference; this was used throughout all preprocessing steps before the data were re-referenced to the linked mastoids (at TP9/TP10) to facilitate a comparison with the P3 and Oddball literature. Horizontal electro-oculographic activity was recorded using bipolar electrodes placed 1 cm to the left and to the right of the outer canthi of the eyes. Vertical electro-oculographic activity was acquired using a similar setup, 1 cm below and above the pupil of the right eye. Offline preprocessing of the EEG and electro-oculographic activity included bandpass filtering in the range 0.2 < Hz < 40.

The artifact correction procedure was based on an independent component analysis (ICA) using the Infomax algorithm in EEGLAB (Delorme and Makeig, 2004). The ICA aims to find a linear representation of non-Gaussian data so that the components are statistically as independent as possible (Hyvärinen and Oja, 2000). Since its introduction to EEG data analysis (Makeig et al., 1996), it has become one of the most popular methods of artifact correction. Components are often manually identified as either EEG sources or artifact sources (related to muscles, eye movements, noise, etc.). In the present study, ICA was used to decompose the 3 (condition) × 12 (subject) data sets independently. To reduce the possibility that preconceived notions would influence how the HMD affected the EEG, the classification of components was conducted in a blind manner. Following this, the weights obtained from all non-artefactual components were used to recompute the scalp-level EEG. In the signal-to-noise analysis, the ICA technique with no artifact correction was contrasted with the more traditional, linear-regression-based correction (Gratton et al., 1983). After the artifact correction, EEG was further analyzed using the Brain Vision Analyzer (Brain Products GmbH, Gilching, Germany). This included segmentation into 1 s epochs that were time-locked to the onset of target stimuli, including 200 ms of baseline activity. The baseline was subtracted before a threshold-based artifact-rejection procedure was applied; this involved removing epochs with an absolute amplitude greater than the maximum of 40 μV or with peak differences greater than 60 μV.

### Peak Detection and Analysis

The windows of ERP peaks were established separately for somatosensory evoked potentials (SEPs) and visual evoked potentials (VEPs) using the grand-average ERPs at the lateral (C3/4) and midline (Fz, Cz, and Pz) channels. When visually scanning the standardized, lateralized activity, the somatosensory N140 was identified as a negative peak in the contralateral sites from 140 to 170 ms, with *T*(11) > 3. The windows of subsequent SEPs and VEPs were based on a visual inspection of the grand-average ERPs and on identification of the local peaks and the zero-crossing points of the grand-average ERP waveform. The resulting windows were averaged over three midline electrodes (Fz, Cz, and Pz) and rounded to the nearest 10 ms interval, yielding three latency windows each for SEPs (P200: 160–310; N200: 280–380; P3: 380–500) and VEPs (P200: 150–260; N200: 200–340; P3: 260–400). Finally, peak-to-peak difference values were calculated for the SEP and VEP N200 and P3 components by subtracting the peak amplitude from the preceding peak value (i.e., N200 – P200 and P3 – N200).

The effects that spatial attention had on ERP peak amplitudes were investigated by conducting full factorial repeated measures ANOVAs for each peak latency window and for each SEP and VEP. ANOVAs were performed using the GLM command of SPSS 23.0. Trials were not included in the analysis if participants responded inaccurately (a difference of 5 or more from the true count). In all ANOVA models, modality relevance (relevant vs. irrelevant), location relevance (relevant vs. irrelevant), channel (Fz vs. Cz vs. Pz), and viewing condition (control vs. VR with hand [VR+H] vs. VR without hand [VR-H]) were set as factors; peak amplitudes or peak-to-peak values were set as dependent variables. As an exception, the ANOVA for the somatosensory evoked N140 was performed on the ERP peak amplitude values obtained at C3/4 site. Also, an additional factor of hemisphere (contralateral vs. ipsilateral) was included in this model because the early somatosensory activity was expected to be stronger at the central sites contralateral to the stimulus side. Visual N1 was not analyzed with ANOVA because there was no control for the lateral eye movements other than asking people to keep their gazes focused on the fixation cross. Peak amplitudes were used as the predicted values, both for early SEPs (N140 and P200) and for VEPs (P200), whereas peak-to-peak values were used for the analysis of subsequent potentials (N2 and P3). Follow-up ANOVAs were conducted separately for each viewing condition in case significant effects of viewing condition or attention were found. Whenever required, Greenhouse-Geisser adjustments for degrees of freedom were performed, and the adjusted *p*-values were reported.

## Results

### Signal and Noise

To compare the efficacy of obtaining relevance-induced ERP components in the VR conditions with hand (VR+H) and without hand (VR-H), we compared the signal and noise for these conditions with the HMD-free control. For each viewing condition, we calculated the noise as the effect size (in root mean square, RMS) of the relevant (vs. irrelevant) modality in the baseline and the signal as the same comparison except within an area of similar length as the noise interval (but within the P3 window). To show how artifact correction affects these comparisons, we provided the same analyses for three common types of corrective procedures: raw (i.e., no correction), regression (based on Gratton et al., 1983) and ICA (Vigário, 1997).

Historically, artefactual data has been removed from the analysis using visual inspection, but as artifacts (such as ocular artifacts, head movements and muscle twitches) tend to cause extreme voltages, it is now more common to apply a threshold for the absolute amplitude or largest difference value within epochs (see Luck, 2005, pp. 152–170 for the general artifact rejection process). One can assume that if a large percentage of epochs is removed due to crossing said threshold, the data are likely strongly confounded by noise. Similarly, if thresholds are changed to remove no more than a certain proportion of the data, then a high threshold indicates a large quantity of noise.

As shown in **Table 1**, linear regression reduces the number of epochs removed in the artifact rejection and decreases the threshold for removing artifacts. ICA again shows its use (Hyvärinen and Oja, 2000) as a technique to reduce noise, as only 3–5% of trials are removed with a 50-μV absolute threshold, and a threshold of 36–40 μV removes 10% of epochs. More importantly, the HMD was not found to induce noise; in fact, the VR-H and VR+H conditions had fewer trials removed than did the control conditions and generally could use a lower threshold for artifact rejections.

**Table 1:** Artifact rejection.

	Epochs removed (%) above 50 μV			Threshold (μV) removing > 10%		
	Control	VR-H	VR+H	Control	VR-H	VR+H
Raw	43.90 (9.21)	27.79 (6.00)	30.63 (6.22)	87.00 (12.08)	64.67 (5.98)	69.42 (7.42)
Regression	26.23 (8.89)^∗^	19.81 (5.28)^∗^	25.42 (6.79)^∗^	67.92 (11.28)	56.42 (5.55)^∗^	73.50 (14.78)
ICA	5.44 (2.13)^∗∗∗^	3.25 (1.99)^∗∗∗^	2.75 (1.24)^∗∗∗^	39.83 (2.89)^∗∗^	36.67 (2.63)^∗∗∗^	37.58 (1.91)^∗∗^

*Artifact rejection as a function of the artifact correction procedure and the experimental condition: Control, VR without hands (VR-H) and VR with hands (VR+H). ^∗^*p* < 0.05, ^∗∗^*p* < 0.01, ^∗∗∗^*p* < 0.001*.

This pattern is to some extent repeated in **Table 2**, which provides the results of the signal and noise ratios (following artifact rejection). In particular, the VEPs, although they were expected to be most affected by the use of the HMD, had a signal that was higher for VR-H and VR+H conditions; the VEPs’ noise was also somewhat lower. For the SEPs, the signal in VR-H was similar to that of the control but was somewhat lower in VR+H; the SEPs’ noise was lower for VR-H and similar for VR+H. Finally, an examination of the topography images showed that HDM had no systematic influence on the ERPs (see Supplementary Figure 1).

**Table 2:** Signal and noise ratios.

VEPs	Signal: RMS (REL-IRR [280, 480])			Noise: RMS (REL-IRR [-200, 0])		
	Control	VR-H	VR+H	Control	VR-H	VR+H
Raw	2.74 (0.39)	3.20 (0.46)	3.56 (0.56)	0.99 (0.20)	0.82 (0.06)	0.80 (0.05)
Regression	2.40 (0.24)	2.99 (0.47)	3.36 (0.55)	0.71 (0.07)	0.75 (0.07)	0.78 (0.11)
ICA	2.08 (0.19)^∗^	2.34 (0.54)^∗^	2.81 (0.41)^∗^	0.64 (0.06)	0.63 (0.07)^∗∗^	0.58 (0.05)^∗∗^

**SEPs**	**Signal: RMS (REL-IRR [350, 500])**			**Noise: RMS (REL-IRR [-200, 0])**		
Raw	2.62 (0.51)	2.67 (0.42)	2.32 (0.39)	0.95 (0.14)	0.74 (0.07)	0.86 (0.08)
Regression	2.32 (0.50)	2.57 (0.42)	2.26 (0.41)	0.68 (0.08)	0.65 (0.07)^∗^	0.97 (0.20)
ICA	2.06 (0.35)	1.93 (0.35)^∗^	2.08 (0.36)	0.65 (0.07)	0.57 (0.10)	0.73 (0.09)^∗^

*Signal calculations using the root mean square (RMS) of the difference between relevant (REL) and irrelevant (IRR) conditions at the P3 interval; the noise (as RMS) for the same conditions around the baseline; and the signal-to-noise ratio (SNR). The table shows the effects of the artifact correction procedure for each of the three conditions of bodily presence: Control, VR without hands (VR–H) and VR with hands (VR+H). The difference of artifact corrections was tested with a series of *t*-tests contrasting the Raw condition to the Regression and ICA. ^∗^*p* < 0.05, ^∗∗^*p* < 0.01, ^∗∗∗^*p* < 0.001*.

### Effects of Spatial Attention on SEPs

**FIGURE 2:**
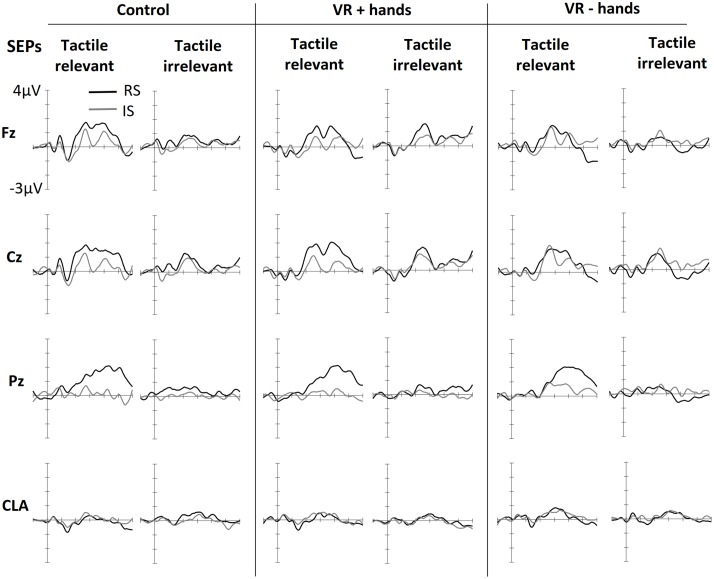
**Grand averaged somatosensory evoked potentials (SEPs) elicited in three conditions of bodily presence (Control, VR+hands, and VR-hands) and obtained at midline (Fz, Cz, and Pz) and contralateral C3/4 sites (CLA)**. Black lines represent SEPs elicited by side-relevant (RS) tactile stimuli, and gray lines represent SEPs elicited by side-irrelevant (IS) tactile stimuli. In each column, the left side shows SEPs elicited by stimuli from the task-relevant modality whereas the right side plots present SEPs elicited by stimuli from task-irrelevant modalities.

**Figure 2** shows ERPs elicited by tactile stimuli at both the midline and lateral sites. The figure is separated into three panels, one for each viewing condition (control, VR+H, and VR-H). The left side of each panel shows the SEPs induced by a tactile stimulus when the tactile modality was relevant—that is, when tactile cues had to be counted. The right side shows the SEPs elicited by task-irrelevant tactile stimuli. First, enhanced contralateral negativity was observed in response to task-relevant tactile stimuli presented on the relevant (vs. irrelevant) side at the range of N140. The effect was similar in all viewing conditions but was present only if tactile stimuli had to be counted. N140 was followed by attentional effects in the subsequent P200, N200, and P3 components; enhanced P200 elicited by a task-relevant tactile target was visible in the control and VR+H conditions but not in the VR-H condition. Interestingly, the same effect was also present in SEPs evoked by modality-irrelevant tactile stimuli if they were presented on the relevant side. However, this cross-modal interaction was mainly present in the VR+H condition. P200 was followed by enhanced attentional negativity in the range of N200. When tactile stimuli had to be counted, the N200 was most enhanced in response to tactile stimuli shown on the irrelevant side. However, if the task was to count visual stimuli, task irrelevant tactile stimuli shown on the relevant side caused the strongest decline, but the effect was again mainly present in the VR+H condition. Finally, a clear P3 component was observed, with the strongest positivity found at the Pz channel. The P3 was clearly present when the tactile stimuli had to be counted but was almost completely absent when the tactile modality was task irrelevant. Contrary to preceding components, no clear differences were found between the viewing conditions.

Supporting the observations of the early negative component, significant main effects of modality relevance, *F*(1,11) = 13.38, *p* = 0.004, $\eta_{p}^{2}$ = 0.55, location relevance, *F*(1,11) = 7.15, *p* = 0.022, $\eta_{p}^{2}$ = 0.39, and hemisphere, *F*(1,11) = 21.99, *p* = 0.001, $\eta_{p}^{2}$ = 0.67, were found in the N140 latency window. The N140 was most enhanced at the sites contralateral to stimulus location and showed stronger negativities in response to modality-relevant tactile stimuli. The significant effect of location relevance indicated that stimuli sent to the irrelevant location resulted in larger N140. Further ANOVAs calculated separately for each hemisphere revealed that modality relevance affected similarly the N140 obtained from the contralateral, *F*(1,11) = 14.83, *p* = 0.003, $\eta_{p}^{2}$ = 0.57, and ipsilateral sides, *F*(1,11) = 7.60, *p* = 0.019, $\eta_{p}^{2}$ = 0.41, but the effect of location relevance was only present on the ipsilateral side, *F*(1,11) = 10.39, *p* = 0.019, $\eta_{p}^{2}$ = 0.41. Although no main or interaction effects of viewing condition were found (*p*s > 0.076), follow-up ANOVAs conducted separately for each viewing condition revealed that modality relevant tactile stimuli resulted in enhanced N140 both in the control and VR+H conditions (*p*s < 0.05, $\eta_{p}^{2}$s > 0.31) but not in the VR–H condition (*p* = 0.09).

In the range of P200, we observed significant effects of location relevance, *F*(1,11) = 9.69, *p* = 0.010, $\eta_{p}^{2}$ = 0.47, and channel, *F*(1,11) = 12.32, *p* < 0.001, $\eta_{p}^{2}$ = 0.53, indicating increased positivity evoked by tactile stimuli presented at the relevant location. As shown in **Figure 2**, somatosensory P200 was similarly affected by location relevance, regardless of modality relevance. The ANOVA results supported this observation, revealing no effect of modality relevance, *F*(1,11) = 1.66, *p* = 0.23. Based on visual inspection, P200 seemed to be most enhanced in the VR+H condition. In contrast, no main or interaction effects of viewing condition were found (*p*s > 0.17). However, conducting the follow-up ANOVAs separately for each viewing condition revealed a significant effect of side in the VR+H condition, *F*(1,11) = 6.66, *p* = 0.026, but not in the control (*p* = 0.06) or VR-H condition (*p* = 0.20).

**FIGURE 3:**
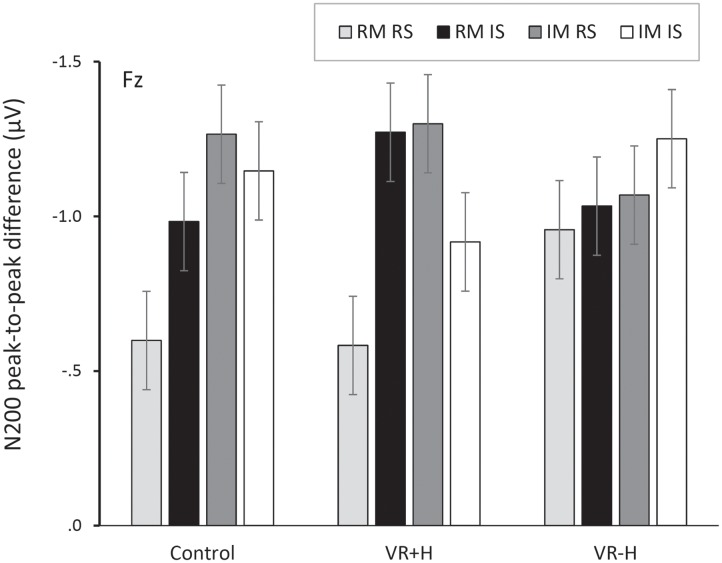
**Peak-to-peak difference between P200 and N200, presented as a function of viewing condition (VR+H vs. VR-H vs. HMD-free control), modality relevance [relevant modality (RM) vs. irrelevant modality (IM)] and location relevance [relevant side (RS) vs. irrelevant side (IS)]**. The figure shows that both modality-relevant and modality-irrelevant tactile stimuli, when presented on the relevant side, are related to enhanced negativity in the range of N200. This attentional negativity occurs both in the control and the virtual-hands conditions.

Investigating the peak-to-peak difference between P200 and N200 revealed significant effects for the modality relevance, *F*(1,11) = 6.48, *p* = 0.027, $\eta_{p}^{2}$ = 0.37; channel, *F*(1,11) = 12.12, *p* < 0.001, $\eta_{p}^{2}$ = 0.52; and modality relevance × location relevance interaction, *F*(1,11) = 19.02, *p* = 0.001, $\eta_{p}^{2}$ = 0.63. This reflects the strongest negativity for modality-irrelevant tactile stimuli presented at the target location. A significant three-way interaction of modality relevance, location relevance, and channel, *F*(2,22) = 4.31, *p* = 0.026, $\eta_{p}^{2}$ = 0.28, revealed that the attentional modulation was particularly strong at Fz and Cz. Finally, a modality relevance × location relevance × channel × viewing condition interaction, *F*(1.86,20.43) = 4.34, *p* = 0.029, $\eta_{p}^{2}$ = 0.28, revealed that the effect of spatial attention on frontal N200 was particularly enhanced in both the control and VR+H conditions; it was not present in the VR-H condition. The same effect is presented in **Figure 3**, which also shows that modality-irrelevant tactile stimuli presented on the relevant side caused N200’s attentional enhancement. This attentional negativity occurs in both the control and VR+H condition but reaches significance only in the VR+H condition, *F*(2,22) = 5.79, *p* = 0.010, $\eta_{p}^{2}$ = 0.34.

In the range of P3, we found a significant main effect of viewing condition, *F*(2,22) = 4.76, *p* = 0.019, $\eta_{p}^{2}$ = 0.30, suggesting that the control and VR+H conditions induced more enhanced P3 activity than did the VR-H condition regardless of spatial attention. No other main effects were found. However, in line with the pattern shown in **Figure 2**, we found that attention was enhanced in P3 only when participants were instructed to count tactile stimuli, *F*(1.26,13.84) = 15.15, *p* = 0.001, $\eta_{p}^{2}$ = 0.58. This target-related positivity was particularly present at central sites, as reflected by a modality relevance × location relevance × channel interaction, *F*(1.27,14.01) = 7.78, *p* = 0.011, $\eta_{p}^{2}$ = 0.41. Viewing condition did not affect the attentional modulation of the P3 component (*p*s > 0.10).

### Effects of Spatial Attention on VEPs

**FIGURE 4:**
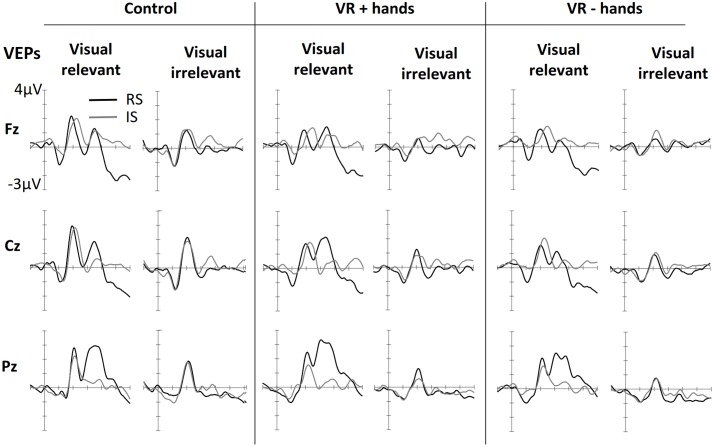
**Grand averaged VEPs elicited in three conditions of bodily presence (Control, VR+hands, and VR-hands) obtained at midline sites**. Black lines represent VEPs elicited by side-relevant (RS) visual stimuli, and gray lines represent VEPs elicited by side-irrelevant (IS) visual stimuli. In each column the left side shows VEPs elicited by visual stimuli when the tactile modality was relevant whereas the right side plots present VEPs elicited by stimuli arising from the irrelevant modality.

**Figure 4** shows ERPs elicited by visual stimuli in three viewing conditions (one panel for each) and measured at three midline sites. The left side of each panel shows ERPs elicited by visual stimuli when the visual stimuli were modality-relevant. The right side shows the VEPs elicited by visual stimuli when the visual modality was irrelevant and tactile stimuli had to be counted. Visual targets elicited larger N1 components at frontal midline sites. The effect was similar for all viewing conditions. N1 was followed by attention-related positivity in the range of 150–260 ms post stimulus. The P200 was most enhanced at posterior sites and in vision-relevant stimulus conditions. No clear difference between viewing conditions was perceived. P200 was followed by attention-related negativity between 200 and 340 ms and was most enhanced at the central site. Similarly to SEPs, modality-relevant visual stimuli presented on the irrelevant side and modality-irrelevant visual stimuli presented on the relevant side resulted in the strongest N200. The pattern was the same in all three viewing conditions, although the decline was strongest in the VR+H condition. Finally, in the range of 260–500 ms, we found a P3 component with strongest positivity at the Pz channel. As with SEPs, the P3 was again more strongly present in visual-relevant stimulus conditions when compared to the visual-irrelevant condition. However, in the control and VR+H conditions, a small P3 component was observed resulting from modality-irrelevant stimuli shown on the irrelevant side.

ANOVAs revealed a significant main effect of modality relevance, *F*(1,11) = 15.67, *p* = 0.002, $\eta_{p}^{2}$ = 0.59, indicating an enhancement in P200 in response to the visual stimuli when the visual modality was relevant. A main effect of viewing condition was also found, *F*(2,22) = 4.31, *p* = 0.026, $\eta_{p}^{2}$ = 0.28, implying that more enhanced P200 amplitudes occurred in the control condition rather than in the VR condition. This effect was accompanied by a significant location relevance × channel × bodily presence interaction, *F*(4,44) = 2.93, *p* = 0.031, $\eta_{p}^{2}$ = 0.21, suggesting that the visual stimuli presented on the cued side resulted in enhanced positivity at the posterior site and that this effect was stronger in the VR+H condition than in the VR-H condition—and was totally absent in the control condition.

No significant main effects of modality relevance or location were found in the N200 range when subtracted from the previous P200 peak, *F*(1,11) < 1.08, *p*s > 0.32. However, main effects were found for viewing condition, *F*(2,22) = 5.36, *p* = 0.013, $\eta_{p}^{2}$ = 0.33, and channel, *F*(2, 22) = 8.05, *p* = 0.002, $\eta_{p}^{2}$ = 0.42. These effects were accompanied by a significant viewing condition × channel interaction, *F*(4,44) = 4.20, *p* = 0.006, $\eta_{p}^{2}$ = 0.28, indicating enhanced negativity at the central sites. This negativity was particularly pronounced in the control condition regardless of the modality or side. Modality relevance was also found to affect N200, but the effect was opposite at the frontal and parietal sites, *F*(2,22) = 17.41, *p* < 0.001, $\eta_{p}^{2}$ = 0.61. Furthermore, we found particularly strong negativities for task-relevant visual stimuli presented on the irrelevant side and modality-irrelevant visual stimuli shown on the relevant side, *F*(1,11) = 5.71, *p* = 0.036, $\eta_{p}^{2}$ = 0.34. This modality relevance × location relevance interaction was most evident in the central and parietal midline electrodes, *F*(2,22) = 16.81, *p* < 0.001, $\eta_{p}^{2}$ = 0.60. Unlike SEPs, viewing condition did not affect this attentional modulation (*p*s > 0.19). Further ANOVAs conducted separately for each viewing condition confirmed this finding reveals a significant three-way interaction of modality relevance, location relevance interaction, and channel in all three conditions (*p*s < 0.12).

Finally, both modality relevance, *F*(1,11) = 11.21, *p* = 0.006, $\eta_{p}^{2}$ = 0.51, location relevance, *F*(1,11) = 11.83, *p* = 0.006, $\eta_{p}^{2}$ = 0.36, and channel, *F*(1.55,17.02) = 6.25, *p* = 0.013, $\eta_{p}^{2}$ = 0.36, were found to affect P3. Presenting a visual, task-relevant stimulus on the relevant side resulted in the strongest positivity, whereas no difference between the relevant and irrelevant sides was found when the tactile stimuli had to be counted, *F*(1,11) = 9.50 *p* = 0.010, $\eta_{p}^{2}$ = 0.46. This interaction effect was accompanied by a three-way interaction between modality relevance, location relevance, and channel, *F*(1.27,13.95) = 5.15, *p* = 0.033, $\eta_{p}^{2}$ = 0.32, indicating that the aforementioned effect was particularly pronounced at the central and parietal midline sites. Similarly to SEPs, viewing condition did not affect the attentional modulation of visually evoked P3 (*p*s > 0.22).

## Discussion

Although the visual enhancement of tactile attention has been demonstrated both with behavioral and neurophysiological recordings (e.g., Longo et al., 2008), the neural underpinnings of body-induced cross-modal interference have remained unclear. Thus, the present ERP study investigated whether viewing one’s real or virtual hands modulates visuo-tactile interaction in endogenous spatial attention at different levels of visual and somatosensory processing. For this purpose, a bimodal oddball task with tactile and visual stimuli was performed under three different viewing conditions: VR with hands, VR without hands, and VR-free control with real hands visible. Based on the previous findings, we assumed the presence of hands would influence attentional modulation of visual and tactile ERPs at both the sensory-perceptual stage (N1, P200) and later, post-perceptual, stages (N200, P3).

### Effect of Bodily Presence on Sensory-Perceptual Processing

As described earlier, attending to a certain spot or stimulus modality potentiates early sensory processing of the attended stimuli (Hillyard et al., 1998a; Hötting et al., 2003) and focusing on the left or right tactile stimuli enhances early visual processing if the visual cue is presented on the attended side (Eimer, 2001). These so-called intramodal, intermodal, and cross-modal spatial modulations were also found in the present study. First, for somatosensory ERPs, an early attentional negativity was obtained at the range of the N140 component. As an example of intermodal attentional modulation, this early negativity was found to be more enhanced in response to modality-relevant tactile stimuli. The amplitude of N140 differed also in terms of stimulus location. Tactile stimuli presented on the irrelevant side resulted in greater negativity than those presented on the relevant side. This effect occurred regardless of the attended modality and was mainly present at the ipsilateral electrodes. In VEPs, the N1 component has been shown to be responsive to task-irrelevant visual stimuli if shown on the attended side. However, in SEPs such an effect does not usually occur, which has been suggested to indicate that, contrary to other modalities, touch can be decoupled from cross-modal attention when being task-irrelevant (Eimer and Driver, 2000). This decoupling was not observed in the current data given that the somatosensory N140 obtained at ipsilateral sites was similarly enhanced in the tactile relevant and irrelevant trials.

More importantly, we found that attentional enhancement of N140 occurred only if the participants could see their real or virtual hands resting on the table. This so-called VET effect has also been found in earlier studies (e.g., Longo et al., 2008; Sambo et al., 2009). However, in these studies the visual body cues were shown to affect spatial attention whereas in the present study seeing one’s hands modulated mainly the intermodal selection (i.e., effects of modality relevance). Although unclear, the contrasting finding may be due to differences in the tasks and target stimuli.

In the range of somatosensory P200, we observed that tactile stimuli presented on the relevant side elicited larger P200 both in tactile and visual relevant trials. This cross-modal interaction in spatial attention was also present in visual P200. Further ANOVAs revealed that the attentional modulation of tactile and visual P200 occurred mainly when virtual or real hands were present, suggesting that the hands made participants more sensitive to both modality-relevant and -irrelevant visual stimuli when presented on the attended side. It is unclear, however, why in VEPs the effect was only present in the VR+H condition but was not found in the control condition, in which participants could also see their hands lying on the table. One reason for this could be the novelty of the 3-D arms, which caused participants to pay more attention to their new limbs than they would to their real hands when seeing them resting on the table. Another explanation is that the vertical distance between tactile and visual cues was slightly different in the VR and HMD-free condition. In correlation to the second explanation, researchers using single-cell recordings from non-human primates have revealed populations of neurons in the parietal region, premotor area, and putamen responding similarly to tactile stimulus presented on the hand and visual stimulus shown near the hand (Graziano and Gross, 1993; for a review, see Reed et al., 2006). Presenting visual stimulus farther away from the hands results in attenuated firing in the neurons (Graziano and Gross, 1995). These body-centered multisensory representations have been suggested to underlie the effects of bodily presence on tactile-spatial selective processing (Sambo et al., 2009), but they can also explain why in the current study the presence of hands enhanced attentional modulation of visual-evoked P200.

### Effect of Bodily Presence on Post-perceptual Processing

Based on the findings of Pavani et al. (2000), we suggested that seeing one’s hands would make the tactile non-targets more distracting, and thus lead to response inhibition indexed by an enhanced N200 component (Azizian et al., 2006). This was exactly what we found. Participants responded with more negative N200 to tactile distractors when they either arose from the same modality or were presented on the same side as the target. This effect was stronger in the VR hands and control conditions than in the VR no-hands condition. Contrary to the expectations, the presence of hands had no effect on visual-evoked N200. Despite this, visual N200 was likewise more sensitive to target-like distractors, indicating an inhibitory role similar to what was observed in SEPs. Thus, although visual target-like distractors also resulted in enhanced response inhibition in VEPs, no extra inhibitory effort was required when (virtual or real) hands were present. The observed discrepancy between visual and tactile N200 is interesting as it shows that visual input of one’s body affects the tuning of tactile spatial attention more strongly than it affects the tuning of visual spatial attention. The finding is important as it confirms and extends previous ERP evidence which suggests that visual body rather than ambient visual information (e.g., the lab environment) is used for remapping tactile stimuli (Sambo et al., 2009). Also, in addition to earlier ERP evidence, here we show how the presence of body affects not only the sensory-perceptual processes but also later executive function.

Finally, we sought to investigate whether bodily presence would influence late attention and memory-related processing. Contrary to preceding components, both visual and tactile P3 were selectively responsible only to target stimuli. Also the viewing condition affected P3, although only in SEPs. The attentional modulation at the P3 range was, however, no different between viewing conditions, suggesting that both target and non-target tactile stimuli resulted in enhanced P3 if the hands were shown. The finding of P3’s selective responsiveness is in line with earlier literature showing that cross-modal interaction in spatial attention is dismissed at the later ranges (Eimer, 2001).

To sum up, the present study was able to show that bodily presence modulates cross-modal spatial attention. This modulation appears mainly between early (N140) and late sensory-perceptual processing (P200) and subsequent inhibition-related processes (N200) but is absent in the later attention- and memory-related P3 component. However, the relatively small sample size and numerous estimated ANOVA models increase the risk of type 1 error; therefore, replications are required to draw more solid conclusions. On the other hand, many recent studies are consistent with the obtained findings. For example, evidence from various behavioral, ERP, and imaging studies has demonstrated that seeing one’s stimulated body part amplifies early somatosensory processing (Macaluso et al., 2000; Longo et al., 2008; Sambo et al., 2009). Our observation that somatosensory processing is affected and visual-evoked potentials are modulated by bodily presence is likewise in line with earlier findings (Graziano and Gross, 1993, 1995). To our knowledge, there is no earlier ERP evidence showing that bodily presence has an amplifying effect on response inhibition in the context of cross-modal spatial attention tasks. Thus, the current findings confirm and extend previous evidence for the role of visual body in endogenous spatial attention.

### Opportunities and Challenges for Future VR-EEG Research

Besides investigating the effect of spatial attention on SEPs and VEPs, we ensured the reliability of ERPs obtained in VR conditions with signal-to-noise-ratio (SNR) analyses. Comparing the SNRs of viewing conditions, we were able to show that the VR conditions had a stronger P3 signal and less noise than what was observed in the HMD-free control condition. The finding was surprising as we assumed that HMD would induce electrical interference in the EEG signal. However, it seems that the higher SNR in the VR conditions could be due to more restricted head movements and ocular artifacts. That is, in the control condition, participants were not wearing the display, which might have encouraged them to move more freely, whereas in the VR condition, their movements were more limited due to the substantial number of cables. Altogether, HMD did not adversely affect the ERPs, which implies that current commercially available VR headsets can safely be used in ERP research without compromising the reliability of EEG recordings.

In future, the EEG-VR research can be utilized in more complex settings. There are, however, some practical limitations that we would like to point out. The HMDs’ immersive visual experience is in part caused by occluding the user’s vision of the peripheral environment. Despite the benefits of this, occluding also causes increased simulator sickness (Moss and Muth, 2011). Such simulator sickness has long slowed down the diffusion of HMD technologies. The new wave of HMDs promises to eliminate the symptoms by expanding the field of view, but some users still feel nausea and have headaches after extended use (Moss and Muth, 2011). Given that EEG experiments normally last for more than an hour, it is possible that such symptoms may appear. However, as a subtype of motion sickness, simulator sickness is highly dependent on the user’s movements (Merhi et al., 2007). Given that, in most cognitive neuroscience experiments, the participants are encouraged to keep still rather than move around, the risk of simulator sickness remains low. In the present study, only one participant reported feeling mild nausea at the end of the experiment. If, however, constant movements are required, the risk of nausea will exist, and that risk should be taken into account when designing the study.

In ordinary experimental paradigms, the potential of VR simulations is nevertheless evident. This has long been acknowledged among cognitive scientists, who have found virtual environments to be particularly useful in research on body-related processes (e.g., Slater et al., 2009; for a review, Blanke, 2012). Virtual versions of the Rubber Hand Illusion have, for instance, revealed how the integration of tactile, visual, and proprioceptive cues is integral to the feeling that our body belongs to us (Sanchez-Vives et al., 2010; Ma and Hommel, 2013). So far, however, the VR-based research on body representation has mainly relied on behavioral and autonomic measures (Sanchez-Vives et al., 2010; Slater et al., 2010; Ma and Hommel, 2015). Additionally, more attention has been paid to how sensory body cues affect body representation than to how perceiving the body influences perception of the extracorporeal world (Harris et al., 2015). Here, we demonstrate how seeing one’s body modifies the cross-modal attentional system and associated electrophysiological features. In future, the same VR-EEG approach can be used to better understand the influences that the body has on more complex cognitive processes, such as self-body relations and out-of-body experiences.

## Ethics Statement

This study was carried out in accordance with the recommendations of guidelines issued by the National Advisory Body on Research Ethics in Finland with written informed consent from all subjects. All subjects gave written informed consent in accordance with the Declaration of Helsinki. The protocol was approved by the Ethics Review Board of Aalto University.

## Author Contributions

VH collected the data, conducted the analyses, and wrote the manuscript. IA designed and created the 3-D environment, programmed the experiment, and assisted in writing the manuscript. GJ contributed to writing the manuscript. NR was involved in the study design and contributed to the writing. MS designed the study and contributed to the analyses, data interpretation, and manuscript drafting. All authors approved the final version of the manuscript.

## Conflict of Interest Statement

The authors declare that the research was conducted in the absence of any commercial or financial relationships that could be construed as a potential conflict of interest.

## Footnotes

1 www.atactech.com/PR_tactors.html

2 www.leapmotion.com
