# Supplementary material for: Manipulating Bodily Presence Affects Cross-Modal Spatial Attention: A Virtual-Reality-Based ERP Study
Source: Front Hum Neurosci. 2017 Feb 22;11:79. doi: 10.3389/fnhum.2017.00079 (PMC5319989; doi:10.3389/fnhum.2017.00079)
Supplement: Supplementary file 1 [file Data_Sheet_1.pdf]

frontiers

Supplementary Material

Manipulating bodily presence affects cross modal attention: A virtual reality based ERP study

Ville J. Harjunen*, Imtiaj Ahmed, Giulio Jacucci, Niklas Ravaja, Michiel M. Spapé

*** Correspondence:** Ville Harjunen: ville.harjunen@helsinki.fi

1 Supplementary Figures

Supplementary figure 1 demonstrates how there was no systematic influence of HDM in the topography images of the three experimental conditions. Supplementary figure 2 shows the physical lab settings (on right) and the corresponding virtual lab (on left).

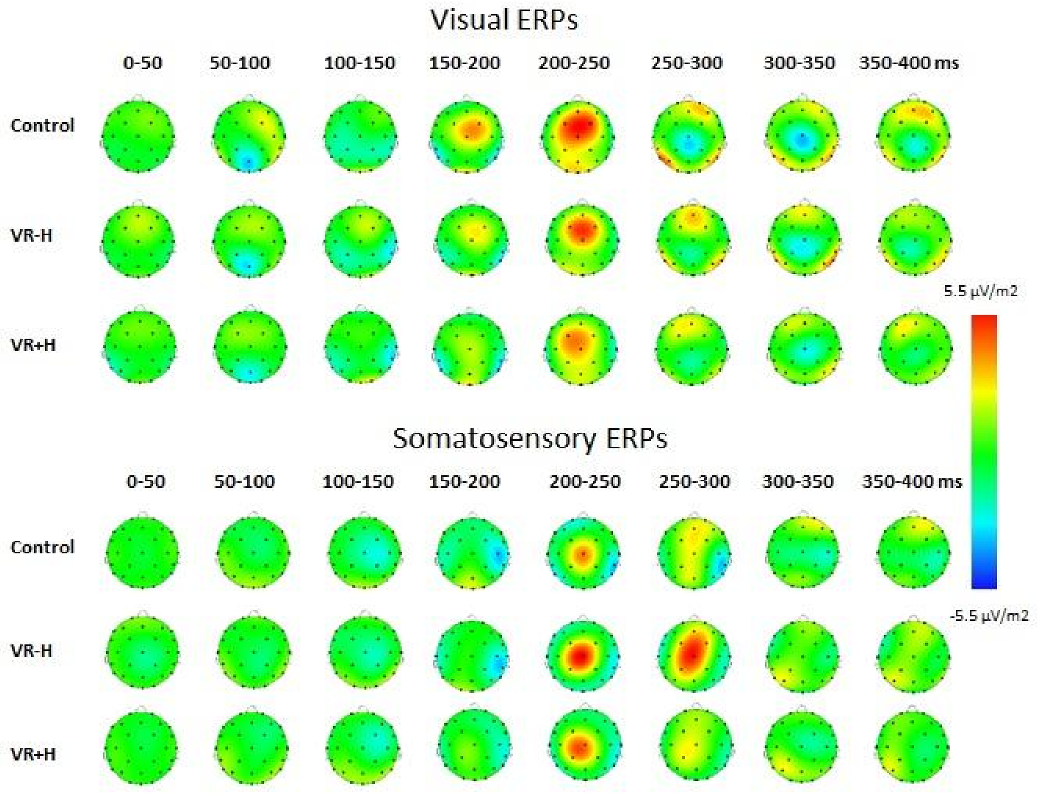

Supplementary Figure 1. Topography images of averaged visual evoked and somatosensory ERPs presented as a function of three experimental conditions (control, VR with hands, and VR without hands) and time.

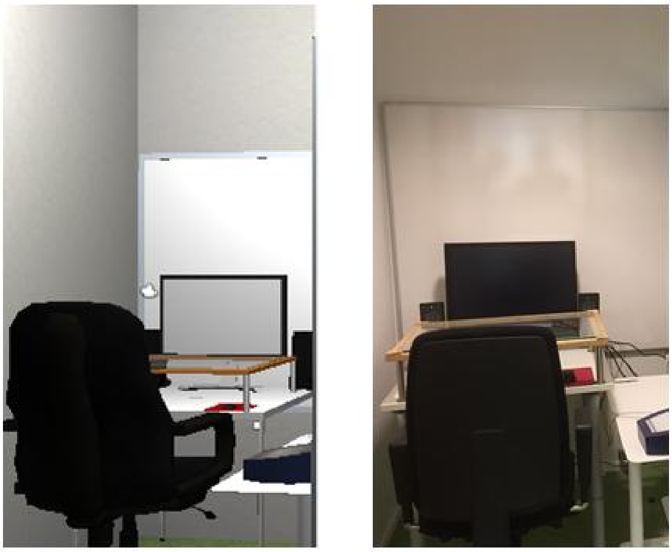

Supplementary Figure 2. Physical lab environment (on the right) and the corresponding virtual environment (on the left).
